# Blockade of L-Type Ca^2+^ Channel Activity Alleviates Oligodendrocyte Pathology following Brain Injury in Male Rats

**DOI:** 10.3390/cimb45050252

**Published:** 2023-05-02

**Authors:** Mohamed A. Al-Griw, Rabia Alghazeer, Haithm W. Ratemi, Mohamed E. Ben-Othman, Refaat Tabagah, Ghalia Shamlan, Mahmmoud M. Habibullah, Afnan M. Alnajeebi, Nouf A. Babteen, Areej A. Eskandrani, Ammar AL-Farga, Wafa S. Alansari

**Affiliations:** 1 Department of Histology and Genetics, Faculty of Medicine, University of Tripoli, Tripoli 13203, Libya; 2 Department of Chemistry, Faculty of Science, University of Tripoli, Tripoli 50676, Libya; 3 Department of Genetic Engineering, Biotechnology Research Center (BTRC), Tripoli 30313, Libya; 4 Department of Clinical Pathology, Faculty of Veterinary Medicine, University of Tripoli, Tripoli 13662, Libya; 5 Division Developmental Biology, Zoology Department, Faculty of Sciences, University of Tripoli, Tripoli 13662, Libya; 6 Department of Food Science and Nutrition, College of Food and Agriculture Sciences, King Saud University, Riyadh 11362, Saudi Arabia; 7 Medical Laboratory Technology Department, Faculty of Applied Medical Sciences, Jazan University, Jazan 45142, Saudi Arabia; 8 Biochemistry Department, Faculty of Science, University of Jeddah, Jeddah 21577, Saudi Arabia; 9 Chemistry Department, Faculty of Science, Taibah University, Medina 30002, Saudi Arabia

**Keywords:** L-type VOCCs, nifedipine, oligodendrocyte, apoptosis, proliferation, oxidative stress

## Abstract

A growing body of studies suggests that Ca^2+^ signaling controls a variety of biological processes in brain elements. Activation of L-type voltage-operated Ca^2+^ channels (VOCCs) plays a role in the development of oligodendrocyte (OL) lineage loss, and indicates that the blocking of these channels may be an effective way to inhibit OL lineage cell loss. For this study, 10.5-day-old male Sprague–Dawley rats were used to generate cerebellar tissue slices. The slice tissues were cultured and randomly allocated to one of four groups (six each) and treated as follows: Group I, (sham control); Group II, 0.1% dimethyl sulfoxide (DMSO) only (vehicle control); Group III, injury (INJ); Group IV, (INJ and treatment with NIF). The injury was simulated by exposing the slice tissues to 20 min of oxygen–glucose deprivation (OGD). At 3 days post-treatment, the survival, apoptosis, and proliferation of the OL lineages were measured and compared. Results: In the INJ group, there was a decrease in mature myelin basic protein+ OLs (MBP+ OLs) and their precursors, NG2^+^ OPCs (Nerve-glia antigen 2+ oligodendrocyte precursor cell), compared with controls. A significant elevation was observed in the NG2^+^ OPCs and apoptotic MBP^+^ OLs as confirmed by a TUNEL assay. However, the cell proliferation rate was decreased in NG2^+^ OPCs. NIF increased OL survival as measured by apoptosis rate in both OL lineages and preserved the rate of proliferation in the NG2^+^ OPCs. Conclusions: Activation of L-type VOCCs may contribute to OL pathology in association with reduced mitosis of OPCs following brain injury as a strategy to treat demyelinating diseases.

## 1. Introduction

Increasing evidence suggests that ischemic brain injury correlates with long-term neurological problems resulting in part from oligodendrocyte (OL) lineage damage and a loss of appropriate myelination [1]. CNS myelination disturbance is a central feature experienced in conditions ranging from ischemia, periventricular leukomalacia, and cerebral palsy in infants [2] to stroke and multiple sclerosis in adults [1,3]. These resulting conditions can leave affected individuals severely handicapped with little or no recovery prospects, yet no specific pharmacotherapies presently exist [4,5,6].

The various cellular brain elements are separately under ischemic attack [7]. The types of glia which are most vulnerable to injury are those of the OL lineages [8]. The precise underlying mechanisms that are involved in ischemic brain injury-induced OL loss are not fully understood. However, in recent years, studies suggest that OL lineages are vulnerable to glutamate excitotoxicity, reactive oxygen species (ROSs) with oxidative stress, release of nitric oxide, and elevated Ca^2+^ influx [9]. Brain ischemia can cause elevated glutamate levels in the extracellular space [10] largely due to the reversal of the glutamate transporters [7,11]. Glutamate is also released via cystine-glutamate anti-porter activity and vesicular release after depletion of ATP [12]. High amounts of glutamate and the resultant sustained activation of Ca^2+^-permeable ionotropic glutamate receptors are involved in subsequent OL death [2,13].

Ca^2+^ homeostasis is essential for the development and survival of many cell types including glial cells of the CNS [14]. The influx of Ca^2+^ through store-operated and voltage-operated Ca^2+^ channels (VOCCs) play an essential role in many cellular processes, such as cell migration and proliferation [15]. Immunocytochemical study showed that the expressions of L-, N-, and R-type VOCC channels in OL lineage cells stem from different brain regions [14,15]. Several processes are regulated by L-VOCCs, such as migration of OL precursor cells (OPCs) [16]. Ca^2+^ dyshomeostasis is related to CNS pathophysiology [14]. Evidence suggests that there is a relationship between excessive Ca^2+^ ion influx and cell death in various cell types [17]. Studies using optic nerve models, in which injury is induced by hypoxia–ischemia, showed that VOCCs participate in the cellular damage process [18]. In cells within the OL lineage, OPCs are extremely vulnerable to prolonged and excessive intracellular Ca^2+^ ion influx resulting from channel and pump dysregulation [16], or from Ca^2+^ that is released from internal stores, which alters the regulatory mechanisms of Ca^2+^. This leads to an inappropriate stimulation of Ca^2^⁺-dependent enzymes and processes and metabolic derangements, and triggers necrosis or apoptosis [15].

Damage to OL lineages results in a loss of appropriate myelination [19]. However, myelinating OL can repair myelin after injury-induced demyelination, and myelin repair derives from recruitment and differentiation of an endogenous population of OPCs [20,21,22,23,24]. The lineage of OLs traverses many distinct steps from OPC expressing a characteristic set of markers, including platelet-derived growth factor α receptor (PDGFαR) and the proteoglycan Nerve-glia antigen 2^+^ (NG2), to mature OLs expressing the myelin genes: myelin genes such as myelin-associated glycoprotein (MAG), myelin oligodendrocyte glycoprotein (MOG), myelin basic protein (MBP), 2′-3′-cyclic nucleotide 3′ phosphohydrolase (CNP), and proteolipid protein (PLP) and their product proteins [20,21,22]. In this study, we hypothesized that brain ischemia would activate L-type VOCCs and that treatment with nifedipine (NIF), a blocker of L-type Ca^2+^ channels, would minimize OL lineage pathology in an ex vivo model system of rat cerebellum. Our results indicate that blocking the L-type VOCC activity reduced MBP+ OL loss. This protection also correlated with the enhancement of survival and proliferation OL precursors (NG2^+^ OPCs). Our findings may provide insight into a pharmacological approach to prevent demyelinating diseases characterized by OL lineage loss.

## 2. Materials and Methods

### 2.1. Ethical Statement

Animal experiments were carried out in accordance with the regulations of the Research Ethics Committee University of Tripoli, Tripoli, Libya. Approval number (ref. BEC-BTRC 29-2020).

### 2.2. Animals and Housing Conditions

Male Sprague–Dawley rats (*n* = 24), aged 10.5 days old and weighing 17.1 ± 1.23 g, were obtained from the Faculty of Sciences at the University of Tripoli, Tripoli, Libya. Animals were maintained at an ambient temperature with a 12 h light/dark cycle. Animals were provided free access to a standard diet and water.

### 2.3. Preparation of Cerebellar Slice Tissues

Methods were based on previously published protocols [25,26,27]. In brief, male, 10.5-day-old Sprague–Dawley rats (*n* = 24) weighing 17.1 ± 1.23 g were used to generate cerebellar slice tissues. The animals were sacrificed, and the cerebellum was dissected and transversely sliced at a thickness of 300 µm on a vibratome (Leica, Germany). Eight to ten tissue slices per rat brain (6 animals per each experimental group) were carefully transferred onto humidified 1.0 µm pore size cell culture inserts (Millipore, Falcon, Bristol, UK) and placed in a 6-well plate (Falcon, Bristol, UK). The slice tissues were kept in one ml of serum-based medium (50% minimum essential medium Eagle (MEME, Sigma, Bristol, UK), 25% HBSS (hanks balanced salt solution, Invitrogen, Bristol, UK), 20% normal horse serum (Invitrogen), 4.6 mM, (*v*/*v*) L-glutamine (Sigma), 21 mM (*v*/*v*) D-glucose (Fisher Scientific, Bristol, UK), 1% penicillin/streptomycin solution (Invitrogen), 4.2 µM (*v*/*v*) L-ascorbic acid (Aldrich-Sigma, Schnelldorf, Germany), and 11 mM (*v*/*v*) NaHCO_3_ at pH 7.2–7.4) in a humidified aerobic incubator (5% CO_2_) at 37 °C for 3 days. The culture medium was replaced with serum-free medium supplemented with 0.3% B27 growth supplement (Invitrogen).

### 2.4. Induction of Cerebellar Slice Tissue Injury

Cerebellar slice tissue injury was simulated by exposing 3-day-old slice tissue cultures to 20 min oxygen–glucose deprivation (OGD) insult. Briefly, the cultured tissues were transferred into filter-sterilized, deoxygenated glucose-free culture medium for 20 min in an anaerobic airtight chamber with a mixture of 95% N2/5% CO_2_ gas flow, temperature maintained at 37 ± 0.5 °C. After OGD insult, the cultures were washed at least three times with fresh oxygenated culture medium containing 5 mg/mL D-glucose and supplemented with 0.3% B27 and returned to their culture conditions under normoxic atmosphere (5% CO_2_) at 37 °C. Non-OGD treated tissue cultures (sham and vehicle control cultures) were maintained for the same time under normoxic atmosphere (5% CO_2_) at 37 °C. To mimic in vivo conditions, the cultures were incubated for 3 days before being fixed for analysis.

### 2.5. Experimental Design

The tissue culture slices were randomly allocated to one of 4 groups (6 each) and treated as follows: Group I (sham control), the cultured tissue slices were cultured under normoxic conditions at 37 ± 0.5 °C at the time points corresponding to those in other experimental groups; Group II (vehicle control), the cultured tissue slices were treated with vehicle alone (0.1% DMSO); Group III (injury; INJ), the cultured tissue slices were cultured and subjected to 20 min of OGD insult in an atmospheric perfusion airtight chamber (95% N2/5% CO_2_) gas flow at 37 ± 0.5 °C. After OGD treatment, the cultures were washed three times with normal medium before returning them to normoxic atmosphere for 3 days of reperfusion; Group IV, (INJ and treatment with blocker NIF), cultured tissue slices were subjected to 20 min of OGD insult and then were treated with the blocker NIF (10 μM). The blocker was added to culture medium 20 min after the OGD end and maintained in the culture medium for 60 min. Appropriate control groups were performed at the corresponding time points with this blocker to test their cytotoxicity.

### 2.6. Cell Viability Assay

Cell viability was assessed using a commercial kit (Molecular Probes, Invitrogen, Darmstadt, Germany) according to the manufacturer’s instructions. Briefly, cultured brain tissues were incubated in the presence of a solution containing 2 µM calcein-AM (Cal, green) and 4 µM ethidium homodimer-AM (EthD-1, red) at 37 °C for 20 min and fixed in paraformaldehyde (4%) (Sigma, Germany) in PBS (Oxoid, Germany) for 10 min at room temperature.

### 2.7. TUNEL Assay

DNA fragmentation was assessed using a commercial kit (Chemicon ApopTag Fluorescein in Situ Apoptosis Detection kit, S7110 Sigma-Aldrich, UK) according to the manufacturer’s instructions. Tissue sections were incubated with TDT enzyme for one hour at 37 °C in the dark and the reaction was terminated with a stop/wash buffer. Following washing, sections were stained with DAPI (4,6-Diamidino-2-Phenylindole) (Vector Laboratories, Bristol, UK) to label nuclei. To identify the identity of the TUNEL-positive cells, co-localization between TUNEL staining and MBP or NG2 immunostaining was determined.

### 2.8. Immunocytochemistry

For immunocytochemical analysis, cerebellar slice cultures were fixed in 4% PFA in PBS for 60 min at room temperature. Following three washes with PBS, the slices were mounted onto glass slides and blocked for 1 h in a serum-blocking solution containing 10% normal goat serum (MP Biomedical, Bristol, UK) and 0.25% Triton 100-X (Sigma, UK) in PBS. Primary antibodies were diluted in PBS and incubated for 24 h at 4 °C in the dark. The slides were washed three times for 5 min each with PBS and incubated with secondary antibodies diluted in PBS for 2 h at room temperature in the dark, followed by three washes in PBS. The primary antibodies used were MBP (myelin basic protein) (1:200 in PBS; MBL), NG2 (Nerve-glia antigen 2+) (1:300 in PBS; Millipore), and BrdU (1:1000 in PBS; Abcam, Bristol, UK). The secondary antibodies used for immunocytochemistry were Alexa Fluor^®^ 633 and 488 (1:100; Invitrogen, Darmstadt, Germany).

### 2.9. Cell Proliferation Assay

Cell proliferation was measured by BrdU (5-bromo-2′-deoxyuridine, Aldrich-Sigma, Gillingham, UK) assay [28]. Cultured tissue slices were incubated in 20 µM BrdU-containing medium for 3 or 24 h prior to fixation. The fixed cultures were incubated with 1N HCl for 10 min on ice followed by a 10-min incubation with 2N HCl at room temperature and placed in an incubator at 37 °C for 20 min. Acidic conditions were neutralized by placing the slides in borate buffer (0.1 M, pH = 8.5) for 12 min. Slides were washed three times for 5 min each with PBS and 1% Triton 100-X and permeabilized in a solution containing PBS (1 M) with 1% Triton 100-X, glycine (1 M), and normal goat serum (5%) for 1 h. The slides were then incubated overnight with anti-BrdU mono-antibody (eBioscience, Oxford, UK) at a dilution of 1: 50 in PBS followed by DAPI staining of the nuclei.

### 2.10. Cell Quantification

Using confocal microscopy, tissue sections were scanned, imaged, and analyzed. Cell scoring was performed as previously described by Al-Griw et al. (2021). In brief, cell counts were performed across a minimum of nine predetermined grid sections outlined by hand using imageJ (Grid size, 400 × 300 µm; Counting frame, 25 × 25 µm; z-depth 50 µm) at 40× or 63× magnifications in 8 to 10 randomly selected fields per slice with three slices per animal per condition.

### 2.11. Statistics

The data were analyzed using GraphPad Prism software (version 7.0). All data were presented as the mean ± SEM for at least five independent experiments, each performed in triplicate. Normality was determined by the computerized Kolmogorov–Smirnov test. Intergroup comparison of normally distributed data was performed with a Student’s paired *t*-test and multiple comparisons were made using a one-way ANOVA followed by a post hoc test for multiple comparisons and Dunnett’s multiple-comparisons tests to detect pairwise intergroup differences. *p* < 0.05 was considered statistically significant.

## 3. Results

### 3.1. L-Type Ca^2+^ Channel Inhibitor NIF Preserves Cell Viability following Brain Tissue Injury

**Figure 1:**
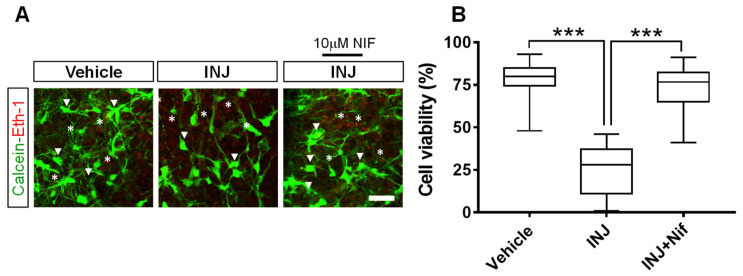
NIF enhances neural cell viability following injury. Tissue sections representing controls, INJ alone, or treatment with NIF. (**A**) Immunofluorescent images of viable cells (calcein-AM (Cal) green, white arrow heads) and nuclei of dead cells (ethidium homodimer-AM (EthD-1) red, asterisks). (**B**) Quantification of cell viability per cubic micrometer. Data are presented as the mean ± SEM of 8 to 10 slices/animal with 6 animals per experimental group. (***) indicates *p* < 0.001.

Here, we identified the effect of L-type VOCC inhibition on cell viability after brain tissue injury. The results indicated the presence of viable cells (Cal, green, white arrow heads) and dead cells (EthD-1, red, asterisks) in all experimental groups (Figure 1A). Specifically, we found that cell viability was significantly lower (*p* < 0.001; Figure 1B) in the INJ group (25.3 ± 3.49%) compared with the sham group (78.95 ± 1.75% per cubic micrometer), and the L-type Ca^2+^ inhibitor, NIF (10 μM), preserved cell viability (72.47 ± 2.63%, *p* < 0.0001; Figure 1B), whereas treatment with vehicle alone showed no effect when applied under controlled conditions.

### 3.2. L-Type Ca^2+^ Channel Inhibitor NIF Minimizes Apoptosis following Brain Tissue Injury

**Figure 2:**
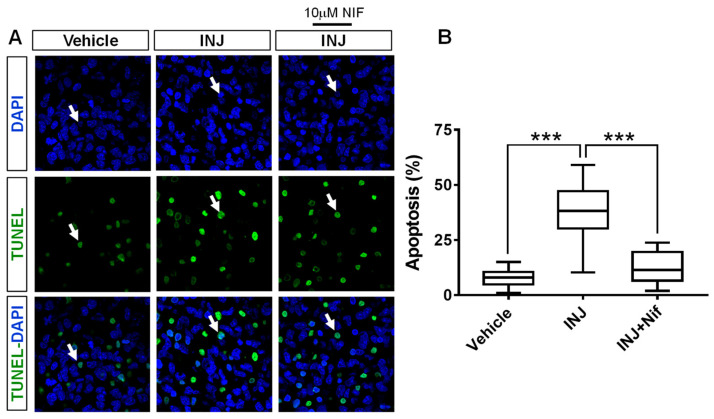
NIF reduces apoptosis following brain tissue injury. Tissue sections representing controls, INJ alone, or treatment with NIF. (**A**) Immunofluorescent images showing apoptotic cells (green, white arrow heads). Scale bar: 20 µm. (**B**) Assessment of apoptosis. Data are presented as the mean ± SEM of 8 to 10 slices/animal with 6 animals per experimental group. (***) indicates *p* < 0.001.

We determined whether NIF treatment-induced protection following brain tissue injury was associated with alterations in apoptosis (pyknotic nuclei). A TUNEL assay in combination with DAPI-nuclei labeling was carried out. The results indicated that in the INJ group, there was increased apoptosis compared with that in the sham group (Figure 2A). Specifically, we found that apoptotic (TUNEL+) cells were increased in number in the INJ group compared with the sham group (38.67 ± 2.17% vs. 9.34 ± 1.04%, *p* < 0.0001; Figure 2B). Treatment with 10 μM NIF markedly reduced apoptosis (12.09 ± 1.29%, *p* < 0.001; Figure 2B).

### 3.3. L-Type Ca^2+^ Channel Inhibitor NIF Restores Mature OL Survival following Brain Tissue Injury

**Figure 3:**
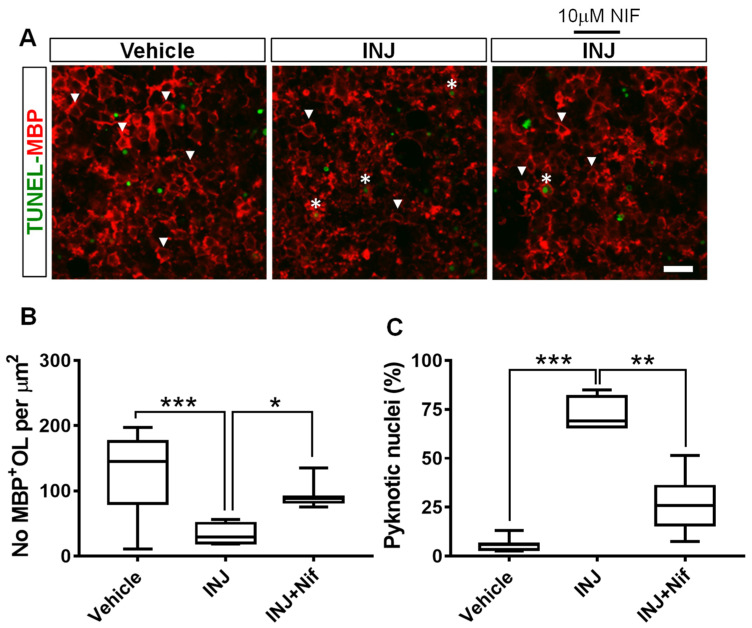
NIF reduces MBP^+^ OL loss following brain tissue injury. Tissues representing controls, INJ alone, or treatment with NIF. (**A**) Immunofluorescent images of MBP^+^ OLs (white arrowheads) and condensed and fragmented nuclei (white asterisks). Scale bar: 20 µm. (**B**) Measurement of MBP^+^ OLs. (**C**) Quantification of apoptotic nuclei among MBP^+^ OLs. Data shown are the mean ± SEM of 8 to 10 slices/animal with 6 animals per group. * indicates *p* < 0.05; ** indicates *p* < 0.01, and *** indicates *p* < 0.001.

Because Ca^2+^ ion influx through L-type VOCCs mediates cell loss in many model systems [29,30], we postulated that blocking L-type VOCC activity may protect maturing OLs. The MBP primary antibody was used for immunocytochemical analysis. No significant shift in morphology and density of MBP^+^ OL nuclei was observed in the absence of injury (Figure 3A, white arrow heads). Uninjured MBP^+^ OLs were dimly fluorescent with oval cell bodies containing three–five nuclear inclusions in a clear cytoplasm. In contrast, the INJ group exhibited a significant MBP^+^ OL loss (Figure 3A,B). The extent of insult rapidly reduced the number of MBP^+^ OLs to 33.24 ± 8.26/mm^2^ compared with 134.8 ± 14.21/mm^2^ for control conditions (*p* = 0.0003; Figure 3B). In contrast, treatment with 10 μM NIF effectively preserved the number of MBP^+^ OLs (90.89 ± 5.77/mm^2^, *p* = 0.0403; Figure 3B).

To determine whether NIF treatment could decrease apoptosis in MBP^+^ OLs in this model system, a TUNEL assay in combination with MBP immunostaining was carried out. Compared with the controls, the INJ group exhibited a significant augmentation in the pyknotic nuclei in the MBP^+^ OLs (Figure 3A, asterisks). Moreover, pyknotic nuclei in the MBP^+^ OLs increased from 6.42 ± 2.67% to 77.95 ± 5.35% (*p* < 0.001; Figure 3B). NIF treatment markedly reduced the density of apoptotic MBP^+^ OLs (36.01 ± 7.35%, *p* = 0.0022; Figure 3C).

### 3.4. L-Type Ca^2+^ Channel Inhibitor NIF Enhances NG2^+^ OPC Survival following Injury

**Figure 4:**
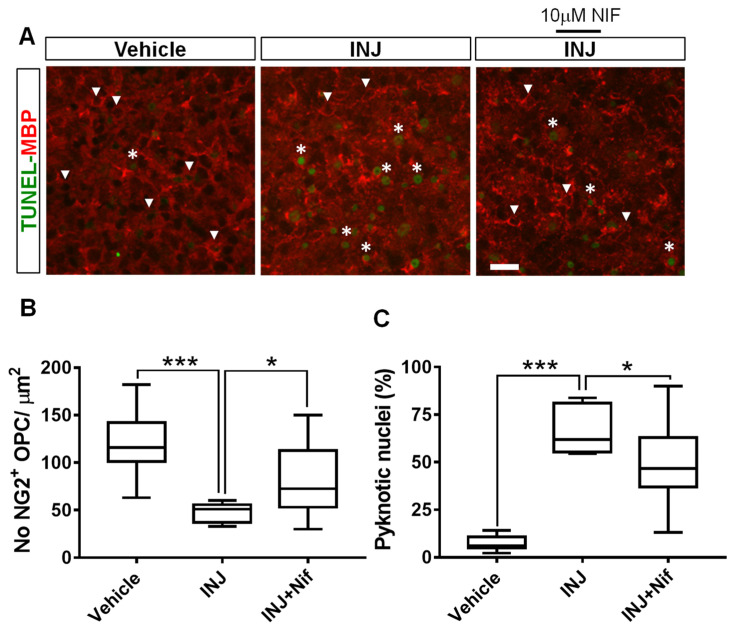
NIF reduces NG2^+^ OPC loss following insult. Tissue sections representing controls, INJ alone, or treatment with NIF. (**A**) Immunofluorescent images of NG2^+^ OPCs (white arrowheads) and pyknotic nuclei (white asterisks). Scale bar: 20 µm. (**B**) Measurement of NG2^+^ OPCs. (**C**) Quantification of apoptosis. Data are shown as the mean ± SEM of 8 to 10 slices/animal with 6 animals per each experimental group. * indicates *p* < 0.05 and *** indicates *p* < 0.001.

OPCs are capable of repairing demyelinated tissue since they can self-renew and mature into myelin-producing OLs cells of the CNS [31]. To determine whether the blocking of L-type VOCC activity affects OPC density and nuclear morphology, we performed a TUNEL assay in combination with immunocytochemical analysis for the early OPC stage-specific marker, NG2. Under control conditions, there was no marked variation in NG2^+^ OPC nuclear morphology or density (Figure 4A, white arrow heads). In the INJ group, only a few individual NG2^+^ OPCs with dim staining were observed (Figure 4A, white arrow heads). The morphology of OPCs conformed to prior descriptions and were characterized by small, irregular, rounded, cell bodies with a few short processes that were highly branched [32]. Furthermore, the INJ group exhibited a significant NG2^+^ OPC loss and a gain in pyknotic nuclei exhibiting brighter TUNEL fluorescence (Figure 4A). The period of insult markedly (*p* = 0.0002) reduced the counts of NG2^+^ OPCs to 47.5 ± 3.64 (Figure 4B) compared with 111.1 ± 9.86 for control conditions. In contrast, treatment with 10 μM NIF effectively preserved the count of NG2^+^ OPCs (83.16 ± 4.83/mm^2^, *p* = 0.03; Figure 4B). Using TUNEL assay, it was shown that pyknotic nuclei among NG2^+^ OPC population increased from 5.63 ± 0.82% to 66.72 ± 4.44% (*p* = 0.012; Figure 4B, asterisks). Ten μM NIF treatment effectively preserved NG2^+^ OPC numbers and restricted numbers of pyknotic nuclei compared to INJ group (48.16 ± 3.47%; Figure 4C).

### 3.5. L-Type Ca^2+^ Channel Inhibitor NIF Preserves NG2^+^ OPC Mitotic Behavior following Injury

**Figure 5:**
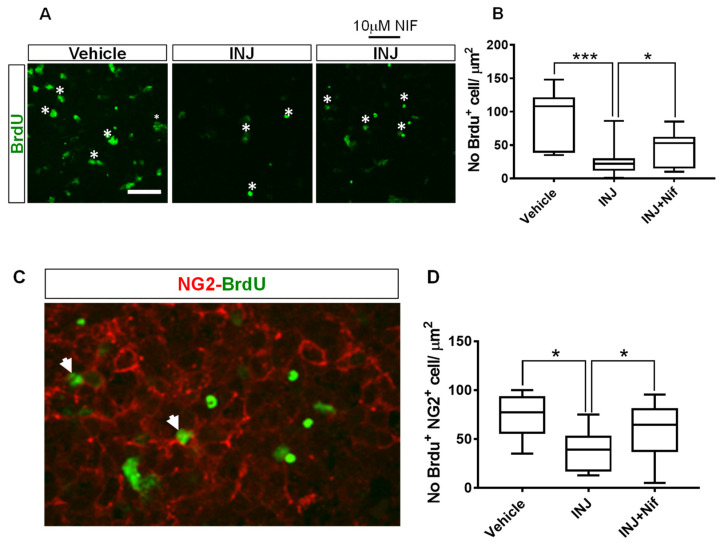
NIF preserves mitotic behavior of NG2^+^ OPCs following insult. Tissue sections representing controls, INJ alone, or treatment with NIF. Immunofluorescence images of (**A**) dividing (BrdU^+^) cells (white asterisks) and (**C**) proliferating NG2^+^ OPCs (white arrowheads). Scale bar: 20 µm. (**B**) Measurement of BrdU^+^ cells. (**D**) Measurement of proliferating NG2^+^ OPCs. Data are presented as the mean ± SEM of 8 to 10 slices/animal with 6 animals per each experimental group. * indicates *p* < 0.05; *** indicates *p* < 0.001.

The effect of NIF on cell mitosis in response to injury was examined. The DNA replication marker, BrdU, was used. The assay showed a significant (*p* < 0.0001; Figure 5B, asterisks) decrease in the INJ group of dividing (BrdU^+^) cells compared with the sham group. The 10 μM NIF treatment markedly preserved cell mitotic behavior compared with the INJ group (*p* = 0.022, Figure 5B).

Because the inhibition of L-type VOCCs with NIF improved OPC survival in this model, we presumed that 10 μM NIF treatment could affect the proliferation of OPCs. To test this, a BrdU assay in combination with immunostaining of an early OPC marker, NG2, was performed. We found that compared with the control, proliferating NG2^+^ OPCs were less abundant in the INJ group and NIF treatment markedly enhanced their density (Figure 5C, white arrow heads). Specifically, in the INJ group, there was a significant (*p* = 0.018) decline in the percentage of BrdU^+^/NG2^+^ cells to 36.64 ± 4.91% compared with 68.05 ± 9.41% for the control group (Figure 5C). The 10 μM NIF treatment significantly protected the mitotic behavior of NG2^+^ OPCs (59.66 ± 7.38%, *p* = 0.036; Figure 5C).

## 4. Discussion

The findings of this study showed that blockade of L-type VOCC activity alleviated OL lineage pathology after ischemic brain tissue. Specifically, the L-type Ca^2+^ channel inhibitor, NIF, reduced apoptosis in MBP+ OLs. In addition, NIF enhanced the mitotic behavior and survival of NG2^+^ OPCs in an ex vivo model system of rat cerebellum. To our knowledge, this is the first demonstration that blocking L-type VOCCs after brain tissue injury protects OL lineages. Hence, the present study highlights the participation of L-type VOCC signaling in the brain in brain injury as the importance of VOCCs in signaling is well known, and suggests a strategy to treat demyelinating diseases.

Cell-based systems have been widely used to investigate the efficacy of a range of agents to treat brain tissue injury [25,33]. Our ex vivo model system of rat cerebellum provides a more suitable CNS environment with a mixed population of different cell types as opposed to studies of homogeneous cell cultures [25,27,28]. Neurodevelopmental deficits constitute a complex interplay of neurons and glia; thus, employing intact brain tissues retains a heterogeneous cell population and the interactions in a more complex multicellular environment may be studied [26]. In the present study, brain tissue injury was induced by subjecting tissue to a brief OGD insult followed by 72 h of reperfusion to mimic the in vivo event [34]. Reperfusion after transient OGD insult results in neural cell loss caused by disruption of cell membrane permeability [34].

The various cellular brain elements are separately under ischemic attack [7]. The precise underlying mechanisms that are involved in ischemic brain injury-induced neural cell loss are not fully understood. However, in recent years, studies suggest that OL lineages are vulnerable to glutamate excitotoxicity, reactive oxygen species (ROSs) with oxidative stress, release of nitric oxide, and elevated Ca^2+^ influx [9]. Brain ischemia can cause elevated glutamate levels in the extracellular space [10,27], largely due to the reversal of the glutamate transporters [7,11,27]. High amounts of glutamate and the resultant sustained activation of Ca^2+^-permeable ionotropic glutamate receptors is involved in subsequent OL death [2,13].

Signaling mechanisms of Ca^2+^ influx and efflux in addition to Ca^2+^ binding proteins play diverse roles in the expression of dependent genes, cell survival, proliferation, and differentiation [35]. Transporters of Ca^2+^, VOCCs, ligand gated Ca^2+^ channels, Ca^2+^ binding proteins, and store-operated Ca^2+^ channels maintain normal Ca^2+^ concentrations and gradients, which are important for normal Ca^2+^ signaling [36]. VOCCs regulate the entry of Ca^2+^ into and from a cell, responding to depolarization of the plasma membrane and converting an electrical signal into a chemical one [36]. VOCCs are multi-subunit structures which include a pore-forming subunit [3]. The pore-forming subunit is encoded by five types of VOCCs and depends on approximately 10 genes [36,37]. VOCC types include high voltage-activated channels (R- N-, L-, and P/Q types), whereas the low voltage-activated channel consists of the T-type.

Increased Ca^2+^ influx through VOCCs plays an essential role in triggering either necrosis or apoptosis [16], which can subsequently activate downstream signaling pathways to exert pro-survival effects [34,38]. A number of preventive modalities have been used to protect cells and tissues after injury or stress and followed by reperfusion, including maintenance of intracellular Ca^2+^ levels [34]. Here, we did not ascertain which specific neural cell type was predominantly affected, the extent of cell loss from neurons or glia, or changes to neuronal and glial interactions. Nonetheless, we found that the L-type VOCC inhibitor, NIF, enhanced neural viability and reduced apoptosis among neural cells. This suggests that blocking L-type VOCC activity may preserve all cell types prone to injury-induced cell death. Our findings illustrate that the L-type VOCC inhibitor, NIF, inhibits apoptosis in the neural cell population in our model system. Therefore, we propose that consistent protection through L-type VOCC inhibition following injury may be attributed to a drug-mediated increase in pro-survival and anti-apoptotic gene expression during the progression of brain tissue injury; however, it may also target excitotoxic and oxidative pathways.

Ischemic brain injury occurs at several distinct sites including axon and glia. Glia are endowed with Ca^2+^-permeable store-operated channels which can be activated through VOCCs, which are activated in response to depolarization of cell membranes, for example, because of an increase in extracellular K^+^ levels [39], all of which determine Ca^2+^ homeostasis [9,14]. As mentioned above, many studies indicate that there is a relationship between excessive Ca^2+^ influx and cell death [40]. Ca^2+^ homeostasis regulation plays an essential role in OL physiology, determining proliferation, migration, differentiation and myelination, damage, repair, or death [14,16,41]. For the normal development of OPCs, modulation of intracellular Ca^2+^ levels by L-type Ca^2+^ channels is important [16]. The types of glia which are most vulnerable to injury are those of the OL lineages [8]. OL lineages appear to be highly vulnerable to prolonged and excessive intracellular Ca^2+^ influx resulting from channel and pump dysregulation [42] or even Ca^2+^ release from internal stores which alters Ca^2+^ regulatory mechanisms. In cells within the OL lineage, OPCs are shown to be extremely vulnerable to prolonged and excessive intracellular Ca^2+^ ion influx resulting from channel and pump dysregulation, or from Ca^2+^ that is released from internal stores, which alters the regulatory mechanisms of Ca^2+^ [16]. Studies demonstrated the presence of N-, L-, and R-type channels in OLs [16,43]. Furthermore, studies in optic nerve models, in which injury is induced by hypoxia-ischemia, have revealed that VOCCs participate in cellular damage [44,45]. Therefore, we hypothesized that simulated brain tissue injury by OGD insult damages OLs lineages by activating L-type VOCCs and that the L-type Ca^2+^ channel inhibitor, NIF, can mitigate OL pathology shortly (20 min) following the insult. Our findings showed that blockade of the L-type Ca^2+^ channel activity after ischemic insult is associated with the preservation of mature OLs (MBP+ cells) in our model system. In addition, the L-type Ca^2+^ channel inhibitor, NIF, minimized apoptosis of the OL lineages. Additionally, we found that the attenuation of L-type VOCC activity by NIF enhanced cell viability and minimized apoptosis in immature OL populations (NG2^+^ OPCs). In addition, NIF preserved the mitotic behavior of NG2^+^ OPCs following injury. Taken together, these findings suggest that inhibition of L-type VOCC activity may impart robust protection and can be effective for the treatment of various neurodegenerative diseases [16]. This suggests that L-type VOCC-mediated Ca^2+^ influx is crucial to OL pathology.

## 5. Conclusions

This work highlights the therapeutic value of blocking L-type VOCC activity, specifically, in preventing or/and reversing OL pathology associated with a number of demyelinating diseases. It is clear from published clinical trials that no effective therapeutic strategy is available in the setting of neonatal brain tissue injury. The occurrence of ischemic brain injury is usually unpredictable and often renders pre-treatment impossible. The efficacy of the selective L-type Ca^2+^ channel blocker NIF as a delaying treatment strategy to treat demyelinating diseases, however, remains unclear. Our findings demonstrate that post-treatment with NIF at a time point immediately after injury attenuated OL loss. This suggests the persistence of L-type VOCC activity after the end of the insult. Because premature infants are generally maintained in a constantly monitored neonatal intensive care unit, blockade activity of L-type VOCCs even within a few minutes post-insult is also feasible to at least minimize brain damage.

## Data Availability

Data generated or analyzed during the current study are available from the corresponding author and included in this published article.
